# Two Prp19-Like U-Box Proteins in the MOS4-Associated Complex Play Redundant Roles in Plant Innate Immunity

**DOI:** 10.1371/journal.ppat.1000526

**Published:** 2009-07-24

**Authors:** Jacqueline Monaghan, Fang Xu, Minghui Gao, Qingguo Zhao, Kristoffer Palma, Chengzu Long, She Chen, Yuelin Zhang, Xin Li

**Affiliations:** 1 Michael Smith Laboratories, University of British Columbia, Vancouver, British Columbia, Canada; 2 Department of Botany, University of British Columbia, Vancouver, British Columbia, Canada; 3 National Institute of Biological Sciences (NIBS), Beijing, People's Republic of China; The University of North Carolina at Chapel Hill, United States of America

## Abstract

Plant Resistance (R) proteins play an integral role in defense against pathogen infection. A unique gain-of-function mutation in the *R* gene *SNC1*, *snc1*, results in constitutive activation of plant immune pathways and enhanced resistance against pathogen infection. We previously found that mutations in *MOS4* suppress the autoimmune phenotypes of *snc1,* and that MOS4 is part of a nuclear complex called the MOS4-Associated Complex (MAC) along with the transcription factor AtCDC5 and the WD-40 protein PRL1. Here we report the immuno-affinity purification of the MAC using HA-tagged MOS4 followed by protein sequence analysis by mass spectrometry. A total of 24 MAC proteins were identified, 19 of which have predicted roles in RNA processing based on their homology to proteins in the Prp19-Complex, an evolutionarily conserved spliceosome-associated complex containing homologs of MOS4, AtCDC5, and PRL1. Among these were two highly similar U-box proteins with homology to the yeast and human E3 ubiquitin ligase Prp19, which we named MAC3A and MAC3B. MAC3B was recently shown to exhibit E3 ligase activity *in vitro*. Through reverse genetics analysis we show that MAC3A and MAC3B are functionally redundant and are required for basal and R protein–mediated resistance in *Arabidopsis*. Like *mos4-1* and *Atcdc5-1, mac3a mac3b* suppresses *snc1-*mediated autoimmunity. MAC3 localizes to the nucleus and interacts with AtCDC5 *in planta*. Our results suggest that MAC3A and MAC3B are members of the MAC that function redundantly in the regulation of plant innate immunity.

## Author Summary

Plants have evolved sophisticated mechanisms to sense and respond to pathogen infection. One robust defense response is mediated by Resistance (R) proteins that recognize specific pathogen-derived effector molecules and initiate signaling cascades in the infected cells to impede pathogen growth. By using mutants with altered defense responses, genetic pathways involved in plant immunity can be studied. Using the model plant *Arabidopsis thaliana*, we previously characterized three proteins in a complex called the MOS4-Associated Complex (MAC) that are integral to plant immune responses and are highly conserved among eukaryotes from yeast to human. We were interested in further studying the role of this protein complex in plants. Here, we report the purification of the MAC and the identification of its components. We further characterized two highly similar MAC proteins in detail, which we called MAC3A and MAC3B. These proteins are functionally redundant and are required for effective resistance responses to several pathogens. Our evidence suggests that the MAC represents a key regulatory node for plant disease resistance.

## Introduction

Plants possess multi-layered defense systems against microbial pathogens. The first layer is governed by a collection of pattern recognition receptors (PRRs) that detect highly conserved features of whole groups of microbes known as pathogen- or microbe-associated molecular patterns (PAMPs or MAMPs) [1]. The second layer is mediated by Resistance (R) proteins, which recognize specific pathogen effectors deployed during an infection. Recognition of effectors or their functions leads to resistance against that pathogen. The majority of cloned *R* genes encode intracellular NB-LRR proteins that contain a central nucleotide-binding site (NB), C-terminal leucine-rich repeats (LRR), and either a Toll/Interleukin-1-receptor-like (TIR) or a coiled-coil (CC) domain at the N-terminus [2]. Signaling through TIR- and CC-NB-LRR proteins is generally streamlined into two pathways [3]; TIR-NB-LRRs signal through ENHANCED DISEASE RESISTANCE 1 (EDS1), PYTOALEXIN DEFICIENT 4 (PAD4) and SENESCENCE ASSOCIATED GENE 101 (SAG101) [4], whereas CC-NB-LRRs signal through NON-RACE SPECIFIC DISEASE RESISTANCE 1 (NDR1) [3]. These pathways later converge and lead to common defense outputs in the infected cells to restrict pathogen growth, including defense gene expression, accumulation of the defense hormone salicylic acid (SA), cell wall strengthening and ion leakage, which in many cases culminate in a form of programmed cell death known as the hypersensitive response (HR) [2]. SA-dependent defense responses are mediated by the protein NON-EXPRESSOR OF PATHOGENESIS RELATED GENES 1 (NPR1) [5]. Mutations in *NPR1* abolish SA-dependent resistance and lead to enhanced susceptibility to pathogen infection [6].

The unique gain-of-function mutant *suppressor of npr1-1, constitutive 1* (*snc1*) suppresses *npr1-*related susceptibility to pathogens in *Arabidopsis* [7]. Both the double *snc1 npr1* mutant and the single *snc1* mutant constitutively express *PATHOGENESIS RELATED* (*PR*) genes and accumulate high endogenous levels of SA, leading to enhanced pathogen resistance. *SNC1* encodes a TIR-NB-LRR R protein homologous to RESISTANCE TO PERONOSPORA PARASITICA 4 (RPP4) [8]. The *snc1* point mutation causes a glutamate to lysine substitution in the linker region between the NB and LRR domains, leading to constitutive SNC1 activation and the constant stimulation of resistance responses even in the absence of pathogens [7],[8]. A suppressor screen to search for novel downstream components of the *snc1*-mediated signaling network led to the identification and characterization of several *MODIFIER OF snc1* (*MOS*) genes as key players in plant immunity [9]–[14]. Like other *mos* mutants described to date, *mos4* alleles suppress all phenotypes associated with *snc1*, including small stature, enhanced resistance to virulent pathogens, constitutive expression of *PR* genes and heightened endogenous levels of SA [12].

MOS4 is the founding member of the MOS4-Associated Complex (MAC), a nuclear protein complex containing the Myb-transcription factor CELL DIVISION CYCLE 5 (AtCDC5/MAC1) and the WD-40 repeat protein PLEIOTROPIC REGULATORY LOCUS 1 (PRL1/MAC2) [12]. *MOS4*, *AtCDC5* and *PRL1* are essential components of plant disease resistance signaling, as knockout mutations in any of these genes render plants more susceptible than wild-type to virulent and avirulent pathogens [12]. AtCDC5 interacts with MOS4 and PRL1 *in planta* [12]. Direct interaction between yeast and human homologs of these proteins has also been shown [15],[16], indicating that the interactions are conserved across kingdoms. Importantly, homologs of AtCDC5, MOS4, and PRL1 have been isolated several times as components of a protein complex in yeast and human known as the Nineteen Complex (NTC) [17]–[25]. This complex, named after the E3 ubiquitin ligase Precursor RNA Processing 19 (Prp19) [17],[19], may facilitate spliceosome assembly [26], in addition to having roles in DNA repair [27]–[29] and cell-cycle progression [30],[31].

Proteomic analyses in yeast and human consistently identify Prp19, CDC5, Spf27/hMOS4 and PRL1 together, suggesting that these proteins form the core of the NTC. Several other proteins, including small nuclear ribonucleoproteins (snRNPs) and RNA-binding proteins, also associate with this core [18],[20],[24],[25]. Based on this, we hypothesized that the *Arabidopsis* MAC must contain more components. Here we report the immuno-affinity purification of the MAC using *mos4-1* complementing transgenic lines expressing HA-tagged MOS4, followed by identification of its components through mass spectrometry (MS). Two of the identified proteins are 82% identical to each other at the amino acid level and share sequence homology with Prp19, which we named MAC3A and MAC3B. Like Prp19, these proteins contain a highly conserved U-box domain [32], and MAC3B was recently shown to exhibit E3 ubiquitin ligase activity *in vitro* [33]. Immunoprecipitation (IP) of MAC3A followed by western blot analysis using an anti-AtCDC5 antibody confirmed that MAC3 is indeed part of the MAC. Reverse genetics analysis revealed that while loss-of-function *mac3a* and *mac3b* single mutants do not display any aberrant phenotypes, double mutant *mac3a mac3b* plants are compromised in basal and R-mediated signaling, and are able to suppress the autoimmune phenotypes associated with *snc1* to the same level as *mos4-1.* This suggests that MAC3A and MAC3B function redundantly in basal and R-mediated defense. Our findings reveal the conserved nature of the MAC and the redundant roles of MAC3A and MAC3B in the regulation of immune responses in plants.

## Results

### Identification of MAC proteins

**Figure 1:**
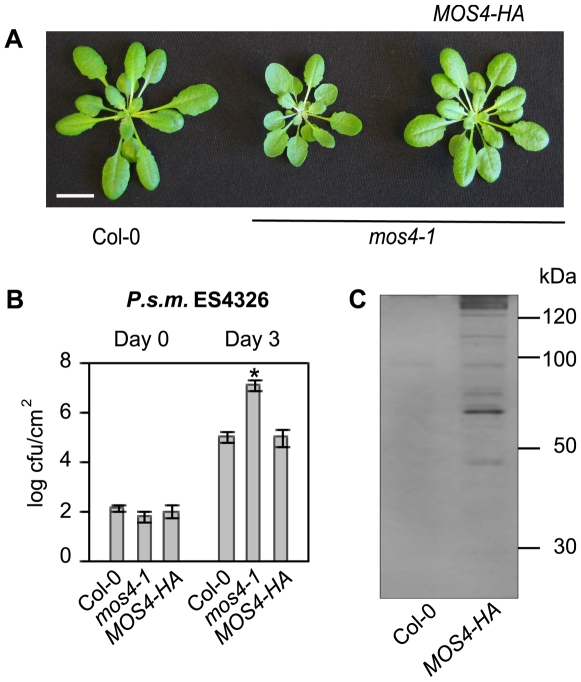
MOS4-HA associated proteins. (A) Full-length genomic *MOS4-HA* driven by its native promoter complements *mos4-1* morphology. Soil-grown plants were photographed 4 weeks after germination. Size bar represents 1 cm. (B) *MOS4-HA* complements *mos4-1* related enhanced susceptibility to *P.s.m.* ES4326, as shown by bacterial growth at 0 and 3 days post-inoculation. Values represent an average of four replicates±SD. An unpaired Student's *t-*test was used to analyze the statistical significance of bacterial growth compared to Col-0. Asterisk indicates P<0.0001. (C) MOS4-HA associated proteins were isolated by affinity purification from total nuclear extracts collected from 100 g of leaf tissue from 3 week old transgenic and Col-0 seedlings. Interacting proteins were eluted, separated by SDS-PAGE on a 12% gel, and silver-stained.

To affinity purify the MAC, full-length MOS4 containing a C-terminal triple hemagglutinin (HA) epitope tag was expressed in *mos4-1* under the control of its native promoter. *MOS4-HA* transformed into *mos4-1* fully complements all *mos4-1* associated phenotypes, including morphology (Figure 1A) and enhanced susceptibility to the virulent pathogen *Pseudomonas syringae* p.v. *maculicula* (*P.s.m.*) strain ES4326 (Figure 1B). This indicates that the MOS4-HA fusion protein functions the same as wild-type MOS4 and thus was used in affinity purification using anti-HA microbeads. As shown in Figure 1C, multiple protein bands were found to specifically associate with MOS4-HA. Any bands in the MOS4-HA lane that were also present in the Col-0 lane were not excised to avoid false positives. MS sequencing of the excised bands identified a total of twenty-four proteins (Table 1, Table S1), including the previously known MAC components MOS4, AtCDC5 and PRL1. Nineteen of the isolated proteins share homology to human and yeast proteins that have been characterized as NTC or NTC-associated proteins (Table 1) [34], indicating that this protein complex is highly conserved throughout eukaryotic kingdoms.

**Table 1:** MOS4-associated proteins identified by mass-spectrometry.

AGI	Protein	*H. sapiens*	*S. cerevisae*	*S. pombe*	NTC Reference
***NTC Core Proteins***					
AT1G09770	AtCDC5/MAC1; R2-R3 Myb transcription factor	CDC5L	Cef1p	Cdc5p	[18], [20]–[25],[43],[51]
AT1G04510	MAC3A; PUB; WD-40 repeat family	hPrp19/SNEV	Prp19p	Cwf8p	[18], [20]–[25],[43],[51]
AT2G33340	MAC3B; PUB; WD-40 repeat family	hPrp19/SNEV	Prp19p	Cwf8p	[18], [20]–[25],[43],[51]
AT3G18165	MOS4; protein-protein interactions	SPF27/BCAS2	- -	Cwf7p	[18],[20],[24],[25],[43]
AT4G15900	PRL1/MAC2; WD-40 repeat family	PLRG1/PRL1	Prp46p/Cwc1p	Prp5p/Cwf1p	[18], [20], [22]–[25],[43],[51]
***Other NTC associated proteins***					
AT1G07360	zinc finger (CCCH-type) family protein; RNA binding	RBM22	Ecm2p	Ecm2p	[20], [23]–[25],[51]
AT1G77180	chromatin protein family	SKIP/SNW	Prp45p	Prp45p/Cwf13p	[20], [22]–[25]
AT2G38770	EMB2765; helicase	KIAA0560	- -	Cwf11p	[24],[25]
AT3G18790	similar to two coiled coil domains of eukaryotic ori	hIsy1	Isy1p/Ntc30p	Cwf12p	[20]–[24]
AT5G28740	transcription-coupled DNA repair-related; RNA processing	hSyf1/XAB2	Syf1p	Cwf3p	[20]–[24],[51]
AT5G41770	cell cycle control crooked neck protein-like; RNA processing	Clf1p/CRN/Syf3	Clf1p/Syf3	Cwf4p	[22],[24],[25],[51]
***Predicted splicing-related proteins***					
AT1G06220	CLO/GFA1/MEE5; translation elongation; nucleic acid binding	U5-116kD	Snu114p	Cwf9p	[20], [22]–[25],[51]
AT1G10580	WD-40 repeat family; nucleotide binding	Cdc40/Dhx38	Prp17p/Cdc40p	Prp17p	[20], [22]–[25]
AT1G15200	protein-protein interaction regulator family	pinin	- -	- -	[24]
AT1G20960	EMB1507; DEAD-box helicase	U5-200kD	Brr2p	Brr2p	[20], [22]–[25]
AT1G32490	EMB2733/ESP3; DEAD-box like helicase	hPrp2/DHX16	Prp2p	Cdc28p	[22],[24]
AT1G80070	SUS2/EMB177; embryogenesis	U5-220kD	Prp8p	Cwf6p	[20], [22]–[25],[51]
AT2G43770	WD-40 repeat family; nucleotide binding	U5-40kD	- -	Cwf17p	[20],[22],[24]
AT5G64270	putative splicing factor similar to RCN1; PP2A regulator	U2-SAP155	Hsh155	Sap155	[18],[22],[25]
***Unrelated proteins/Possible contaminants***					
AT3G15730	PLDα1; hormone response	- -	- -	- -	- -
AT3G20820	LRR family; defense response	- -	- -	- -	- -
AT3G60190	ADL1E/DRP1E/EDR3; GTPase; defense response	DNM1	- -	- -	- -
AT4G19410	putative pectin acetylesterase; carboxylesterase activity	- -	- -	- -	- -
AT5G42080	ADL1A/DRP1A/RSW9; GTPase	DNM1	- -	- -	- -

Nuclear extracts from complementing *mos4-1* plants expressing *PMOS4-MOS4-HA* were immunoprecipitated using anti-HA beads, separated by SDS-PAGE and silver-stained (Figure 1C). The MOS4-HA IP lane was cut into eight pieces, digested, and the proteins contained were analyzed by mass-spectrometry. Sequenced peptides were used as queries in BLAST searches against the 2007 version of the *Arabidopsis* genome. Proteins with strong or partial homology to *Arabidopsis* proteins from other eukaryotes are listed in the columns to the right. Proteins are divided into sections according to their sequence homology to known NTC- or NTC-associated proteins, based on a previous computational organization [34]. Dashes indicate that no proteins with significant homology were identified by BLAST. **Abbreviations:** ADL: *Arabidopsis* dynamin-like protein; BCAS: Breast carcinoma sequence; BRR: Bad response to refrigeration; CDC: Cell division cycle; CEF1: *S. cerevisiae* homolog of cdc5(+); CLF: Crooked neck-like factor; CLO: Clotho; CRN: Crooked neck pre-mRNA splicing factor-like; CWC: Complexed with Cef1p; CWF: Complexed with Cdc5p; DHX: DEAH-box; DNM: Dynamin; DRP: Dynamin-related protein; EDR: Enhanced disease resistance; EMB: Embryo lethal; ESP: Enhanced silencing phenotype; GFA: Gametophytic factor; HSH: Human Sap homolog; ISY: Interactor of Syf; LRR: Leucine-rich repeat; MAC: MOS4-associated complex; MEE: Maternal effect embryo arrest; MOS: Modifier of *snc1*; NTC: Nineteen complex; PLDα: Phospholipase D alpha; PP2A: Protein phosphatase type 2A; PRL/PLRG: Pleiotropic regulating locus; PRP: Precursor mRNA processing; RBM: RNA binding motif; RCN: Roots curl in NPA; RSW: Radial swelling; SAP: Splicing associated protein; SKIP: Ski-interacting protein; SNEV: Senescence evasion factor; SNU: Small nuclear ribonucleoprotein associated; SNW: “SNW” domain; SPF: Spliceosome-associated protein; SUS: Abnormal suspensor; SYF: Synthetic lethality with Cdc40; XAB: XPA-binding protein.

Most of these proteins have not been studied in *Arabidopsis* but are predicted to be involved in pre-mRNA splicing or RNA processing based on their relatedness to NTC proteins. Among others, four predicted subunits of the U5 snRNP, one subunit of the U2 snRNP, and several RNA binding proteins and helicases were identified. In addition, five unrelated proteins were also revealed as potential MAC components (Table 1). These could be novel MAC proteins or they could represent experimental contaminants. Importantly, we identified two proteins, encoded by *At1g04510* and *At2g33340*, with homology (23–24% identity, 41% similarity) to the E3 ubiquitin ligase Prp19, the founding member of the NTC. These proteins, which we named MAC3A and MAC3B, are 82% identical to each other at the amino acid level. An alignment of the amino acid sequences of *Arabidopsis* MAC3A and MAC3B with homologous sequences in several eukaryotes reveals a highly conserved U-box domain at the N-terminus (Figure S1). U-box domains have been shown to exhibit E3 ubiquitin ligase activity in several eukaryotic proteins. In a recent survey of plant U-box proteins, MAC3B was shown to exhibit E3 ubiquitin ligase activity *in vitro* [33], an enzymatic activity that has also been demonstrated in yeast and human Prp19 proteins [35],[36] **.** In addition, these proteins contain a number of C-terminal WD-40 repeats predicted to engage in protein-protein interactions [37].

### Isolation of *mac3a* and *mac3b* loss-of-function mutants

**Figure 2:**
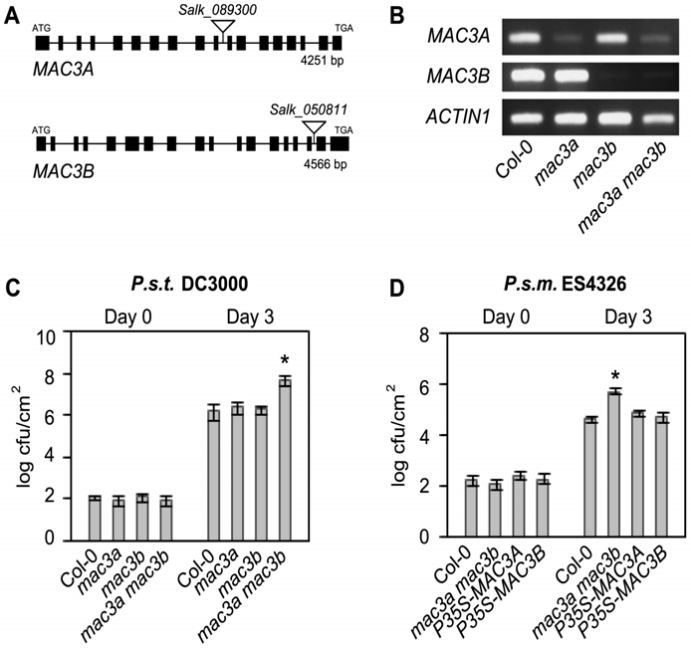
*MAC3A* and *MAC3B* function redundantly in basal defense. (A) Gene structures of *MAC3A* (*At1g04510*) and *MAC3B* (*At2g33340*) showing the position of T-DNA insertions Salk_089300 (*mac3a*) and Salk_050811 (*mac3b*). Lines indicate introns and boxes indicate exons. The location of translation start (ATG) and stop (TGA) codons are found in the first and last exons, as indicated. (B) Expression levels of *MAC3A* and *MAC3B* in Col-0, *mac3a, mac3b,* and *mac3a mac3b* mutants as indicated by semi-quantitative RT–PCR using cDNA–specific primers flanking the T–DNA insertion sites. (C) Growth of *P.s.t.* DC3000 at 0 and 3 days post-inoculation. Values represent an average of four replicates±SD. An unpaired Student's *t-*test was used to analyze the statistical significance of bacterial growth compared to Col-0. Asterisk indicates P<0.001. (D) Growth of *P.s.m.* ES4326 at 0 and 3 days post-inoculation. *P35S-MAC3A-CFP* and *P35S-MAC3B* are expressed in the *mac3a mac3b* background. Values represent an average of four replicates±SD. An unpaired Student's *t-*test was used to analyze the statistical significance of bacterial growth compared to Col-0. Asterisk indicates P<0.0001. Experiments were repeated at least three times with similar results.

Since the MAC3 homolog in yeast and human is an integral member of the NTC and MAC3A and MAC3B were identified as potential MAC components in this study (Table 1), we were interested in testing if the biological function of MAC3 is related to MOS4 through the analysis of knockout mutants. T-DNA insertion mutants were obtained from the Arabidopsis Biological Resource Center (ABRC) [38]. Salk_089300 (*mac3a*) carries an insertion in the intron between exons 10 and 11 of *MAC3A*, and Salk_050811 (*mac3b*) carries an insertion in the intron between exons 16 and 17 of *MAC3B* (Figure 2A). Homozygous lines were identified by PCR-based genotyping. To determine whether the T-DNA insertions affect *MAC3A* and *MAC3B* expression, semi-quantitative RT-PCR was performed using cDNA-specific primers flanking the insertions. As shown in Figure 2B, *mac3a* and *mac3b* both exhibit significantly reduced mRNA expression. Given that MAC3A and MAC3B are 82% identical and single *mac3a* and *mac3b* mutants showed no aberrant morphological phenotypes (Figure S2A), we hypothesized that *MAC3A* and *MAC3B* may function redundantly. When we crossed *mac3a* and *mac3b* to create a double *mac3a mac3b* mutant, plants with a distinct morphology similar to *mos4-1* were observed. These plants are slightly smaller than Col-0 wild type (Figure S2A), have darker leaves, and they flower late (data not shown). Genotyping of the F_2_ progeny confirmed that only these plants were homozygous for both *mac3a* and *mac3b* alleles, indicating that the observed mutant phenotypes co-segregate with both T-DNA insertions and that mutations in both genes are the likely cause of the observed phenotypes.

### MAC3A and MAC3B function redundantly in basal defense

Since mutations in any of the three previously characterized MAC genes *MOS4, AtCDC5* and *PRL1* result in enhanced disease susceptibility, and since the *mac3a mac3b* mutant phenocopies *mos4-1* morphologically, we tested if *MAC3* is likewise required for plant immunity against virulent pathogens. To test whether *MAC3* is required for basal defense responses, *mac3a* and *mac3b* single mutants and *mac3a mac3b* double mutants were infected with a low dose (OD_600_ = 0.0001) of the bacterial pathogens *P. syringae* p.v. *tomato* (*P.s.t.*) DC3000 and *P.s.m.* ES4236 and pathogen growth was assayed after three days. Resistance to *P.s.t.* DC3000 and *P.s.m.* ES4326 was similar in Col-0, *mac3a* and *mac3b* single mutants but was compromised in *mac3a mac3b* double mutant plants, which harboured an over 10-fold higher titer of bacteria compared to Col-0 in both cases (Figure 2C and 2D). This is similar to bacterial growth in *mos4-1* and *Atcdc5-2* (Figure S2B). Importantly, transgenic expression of either *MAC3A* or *MAC3B* cDNA driven by the constitutive *35S* promoter complements *mac3a mac3b* morphology (Figure S3A) and susceptibility to *P.s.m.* ES4326 (Figure 2D). Thus, the enhanced susceptibility phenotype observed in *mac3a mac3b* double mutants is due to the mutations in *MAC3A* and *MAC3B.* Together, these data suggest that *MAC3A* and *MAC3B* play redundant roles in basal defense against virulent pathogens.

### *mac3a mac3b* displays defects in R protein–mediated defense pathways

**Figure 3:**
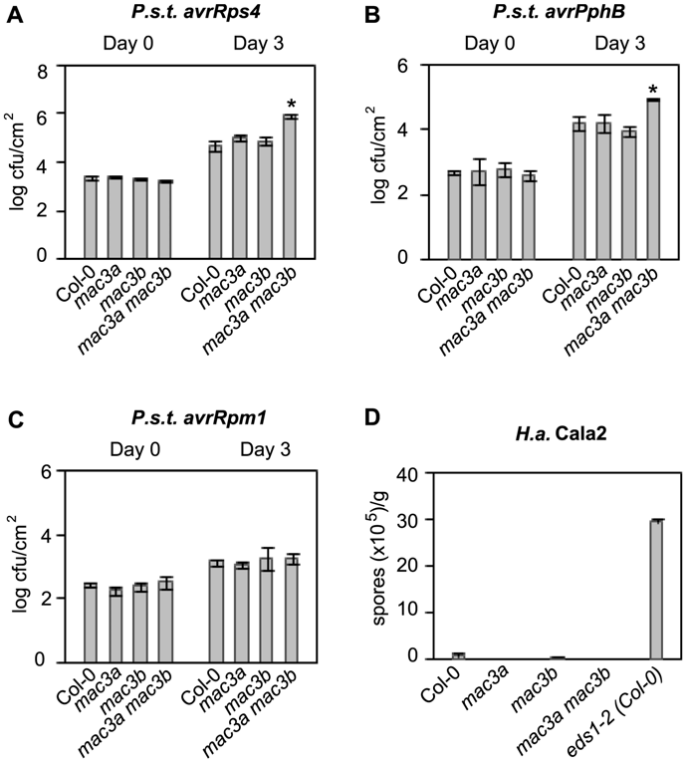
*MAC3A* and *MAC3B* function redundantly in specific R-protein–mediated resistance pathways. (A–C) Growth of the bacterial pathogens *P.s.t. avrRps4, P.s.t. avrPphB,* and *P.s.t. avrRpm1* at 0 and 3 days post-inoculation. Values represent an average of four replicates±SD. An unpaired Student's *t-*test was used to analyze the statistical significance of bacterial growth compared to Col-0. Asterisks indicate P<0.0005. (D) Growth of avirulent *H.a.* isolate Cala2 seven days post-inoculation on 2-week-old seedlings. *eds1-2* introgressed in Col-0 was used as a positive control for pathogen growth. Values represent an average of two replicates of at least 20 plants per genotype±SD. All experiments were repeated at least three times with similar results.

To test whether different R protein-mediated signaling pathways rely on *MAC3*, we challenged *mac3a, mac3b,* and *mac3a mac3b* mutants with pathogens that express specific effectors recognized by distinct R proteins in *Arabidopsis*. Resistance against *P.s.t.* DC3000 expressing either *avrRps4* (recognized by the TIR-NB-LRR R protein RESISTANT TO P. SYRINGAE 4; RPS4) or *avrPphB* (recognized by the CC-NB-LRR R protein RPS5) is compromised in *mac3a mac3b* double mutants but is unaffected in *mac3a* or *mac3b* single mutants, as indicated by an approximate 10-fold increase in bacterial growth in *mac3a mac3b* plants three days after infection compared to Col-0 (Figure 3A and 3B). Conversely, resistance against *avrRpm1* (conditioned by the CC-NB-LRR R protein RESISTANCE TO P.S.M. 1; RPM1) is not impaired in *mac3a mac3b* double mutants, as bacterial growth is comparable to Col-0 (Figure 3C). Resistance against *Hyaloperonospora arabidopsis* (*H.a.*, formerly *H.parasitica*) isolate Cala2 (recognized by the TIR-NB-LRR R protein RESISTANT TO PERONOSPORA PARASITICA 2; RPP2) is also unaffected in *mac3a*, *mac3b*, or *mac3a mac3b*, as indicated by the level of conidiospores collected one week after infection, which was similar to Col-0 (Figure 3D). Together, these data further support redundancy between *MAC3A* and *MAC3B*, and suggest that these proteins are required for signaling pathways mediated by specific TIR- and CC-NB-LRR R proteins.

### *mac3a mac3b* suppresses autoimmune phenotypes associated with *snc1*

**Figure 4:**
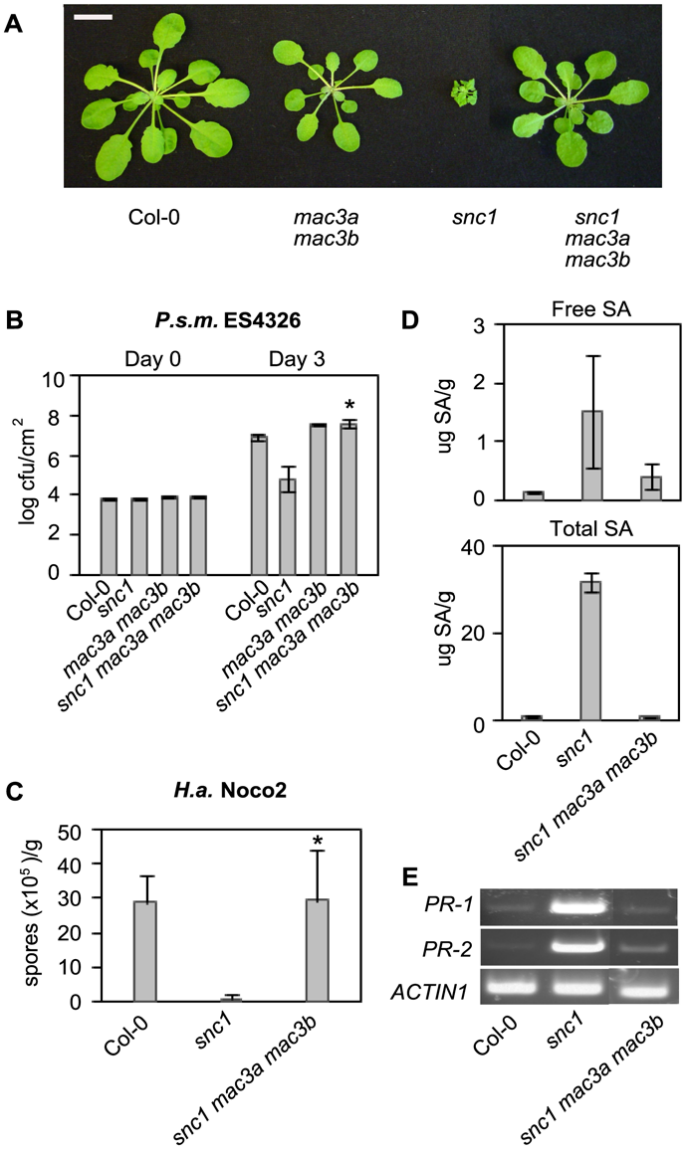
Suppression of *snc1-*associated phenotypes by *mac3a mac3b.* (A) Morphology of Col-0, *mac3a mac3b*, *snc1,* and *snc1 mac3a mac3b* plants. Soil-grown plants were photographed 4 weeks after germination. Size bar represents 1 cm. (B) Growth of *P.s.m.* ES4326 at 0 and 3 days post-inoculation. Values represent an average of four replicates±SD. P values were calculated using an unpaired Student's *t*-test comparing the mean bacterial growth in *snc1* to that in *snc1 mac3a mac3b*. Asterisk indicates P<0.0005. (C) Growth of the *H.a.* isolate Noco2 seven days post-inoculation on 2-week-old seedlings. Values represent an average of 4 replicates of at least 20 plants per genotype±SD. P values were calculated using an unpaired Student's *t*-test comparing oomycete growth in *snc1* compared to that in *snc1 mac3a mac3b*. Asterisk indicates P<0.01. (D) Free and total SA was extracted from leaf tissue from 3-week-old soil-grown plants and analyzed by HPLC. Values represent the average of 4 replicates±SD. (E) Semi-quantitative RT–PCR of the pathogenesis-related genes *PR-1* and *PR-2*. *Actin1* was included as a control. All experiments were repeated at least twice with similar results.

Mutations in *MOS4* or *AtCDC5* suppress *snc1,* indicating that these MAC proteins contribute to *snc1*-mediated resistance [12]. To test if *MAC3* is also part of the *snc1* pathway, we crossed *snc1* with *mac3a mac3b* to obtain a *snc1 mac3a mac3b* triple mutant. Whereas *snc1* plants are of small stature and have dark, curly leaves, the triple mutant does not exhibit *snc1-*like morphology and resembles the *mac3a mac3b* double mutant (Figure 4A). The *snc1* mutant exhibits enhanced resistance to the virulent pathogens *P.s.m.* ES4326 and *H.a.* isolate Noco2 [7]. To test if *snc1*-mediated enhanced resistance is impaired in *snc1 mac3a mac3b* plants, we infected plants with these two pathogens. As shown in Figure 4B, *snc1 mac3a mac3b* triple mutants sustain an approximate 100-fold higher titer of *P.s.m.* ES4326 compared to *snc1* three days after infection, to a level similar to *mac3a mac3b* (Figure 4B). *Agrobacterium-*mediated transformation of *P35S-MAC3A* or *P35S-MAC3B* into *snc1 mac3a mac3b* restored *snc1* morphology (Figure S3B) and resistance to *P.s.m.* ES4326 (Figure S3C). Furthermore, *snc1 mac3a mac3b* mutants are as susceptible to infection by *H.a.* isolate Noco2 as Col-0 plants (Figure 4C), indicating that *mac3a mac3b* completely suppresses *snc1*-related enhanced resistance to these pathogens. In addition to increased resistance, *snc1* plants also accumulate high levels of the defense signaling molecule SA. High performance liquid chromatography (HPLC) analysis of SA extracts collected from Col-0, *snc1*, and *snc1 mac3a mac3b* plants revealed a marked reduction in endogenous free and total SA levels in the triple mutant comparable to the levels in Col-0 (Figure 4D). Semi-quantitative RT-PCR analysis indicated that *mac3a mac3b* also suppresses the expression of *PR-1* and *PR-2*, which are constitutively up-regulated in *snc1* plants (Figure 4E). Together, these data demonstrate that, similar to *mos4-1, mac3a mac3b* completely suppresses *snc1-*mediated autoimmune phenotypes.

### MAC3 localizes to the nucleus

**Figure 5:**
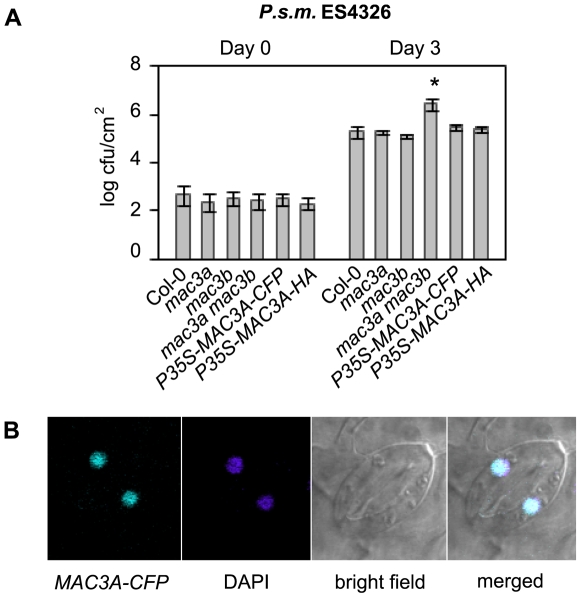
MAC3A localizes to the nucleus. (A) Complementation of *P35S-MAC3A-CFP* and *P35S-MAC3A-HA* in *mac3a mac3b.* Growth of virulent *P.s.m.* ES4326 at 0 and 3 days post-inoculation. Values represent an average of four replicates±SD. This experiment was repeated twice with similar results. An unpaired Student's *t-*test was used to analyze the statistical significance of bacterial growth compared to Col-0. Asterisk indicates P<0.0005. (B) Confocal microscopy was used to examine the localization of *P35S-MAC3A-CFP* in transgenic *mac3a mac3b* plants. Guard cells from a representative line are shown. DAPI was used as a control for nuclear localization.

Several lines of evidence already suggest that MAC3 localizes to the nuclear compartment. First, both MAC3A and MAC3B proteins were isolated by affinity purification from nuclear protein extracts as described above (Table 1). In addition, MAC3A and MAC3B have nuclear localization signals in their protein sequence, as predicted by PSORT (version 6.4; Figure S1). To confirm that MAC3 is nuclear, we created *P35S-MAC3A-CFP* and *P35S-CFP-MAC3B* fusion constructs and transiently transformed onion cells by particle bombardment. In both cases, transformed cells showed exclusive nuclear localization (data not shown), as observed by a fluorescence microscope. To corroborate this data, we created stable *P35S-MAC3A-CFP* transgenic lines in the *mac3a mac3b* background using *Agrobacterium*-mediated transformation. The majority (10/11) of transgenic plants complemented *mac3a mac3b* morphological phenotypes, and all complementing lines showed exclusive nuclear localization of *P35S-MAC3A-CFP* under a confocal microscope. Importantly, resistance to *P.s.m.* ES4326 was restored in *mac3a mac3b P35S-MAC3A-CFP* plants (Figure 5A), indicating proper localization of over-expressed *MAC3A-CFP*. Guard cells from one of these lines are shown in Figure 5B, however nuclear localization was also observed in other tissues including roots (data not shown). Likewise, expression of *P35S-CFP-MAC3B* in complementing *mac3a mac3b* lines (data not shown) also showed distinct nuclear localization (Figure S4). Expression of *P35S-MAC3A-CFP* or *P35S-CFP-MAC3B* in *snc1 mac3a mac3b* also displayed nuclear localization (data not shown), indicating that MAC3A and MAC3B localization is not altered in *snc1*. Together, these data suggest that MAC3 localizes to the nucleus.

While cloning *MAC3B*, we noticed a discrepancy at the 3′ end of the *MAC3B* cDNA sequence. Sequence analysis of both strands of two individually cloned full-length *MAC3B* cDNA constructs confirmed the presence of two cytosines at a position previously shown to contain only one cytosine (Figure S5). This extra cytosine causes a shift in frame and results in an earlier stop codon than the one predicted by The Arabidopsis Information Resource (TAIR8), making the protein 525 amino acids as opposed to the predicted 563 amino acids in length. The corrected cDNA sequence translates into a polypeptide that better aligns at the C-terminal end with MAC3A and Prp19 (Figure S1). This corrected *MAC3B* cDNA sequence has been deposited to GenBank under accession number FJ820118.

### MAC3 associates with AtCDC5 *in planta*

**Figure 6:**
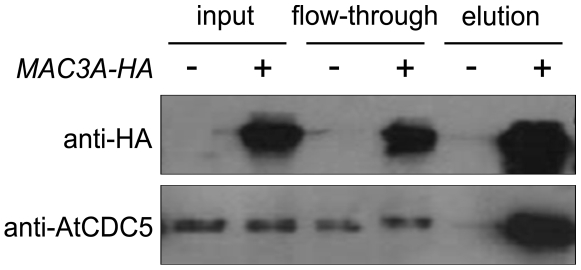
MAC3A-HA associates with AtCDC5 *in planta*. Total nuclear extracts were isolated from a complementing *mac3a mac3b* transgenic line expressing *P35S-MAC3A-HA* (+) and Col-0 (−). MAC3A-HA was immunoprecipitated using anti–HA microbeads. MAC3A-HA and AtCDC5 were detected in the eluted fractions by western blot analysis using antibodies against HA or AtCDC5.

Prp19 interacts with CDC5 in yeast and human cells [15],[39],[40]. Since the MAC seems to be conserved across eukaryotes, we hypothesized that this interaction might also be conserved in *Arabidopsis*. To confirm that MAC3 associates with the MAC, and to corroborate our MS data, we tested whether MAC3 interacts with AtCDC5. To do this, *P35S-MAC3A-HA* tagged fusion constructs were stably transformed into *mac3a mac3b* by *Agrobacterium*-mediated transformation. This fusion protein fully complements *mac3a mac3b* mutant phenotypes and susceptibility to *P.s.m.* ES4326 (Figure 5A) suggesting that it functions the same as the native protein. Total nuclear protein was extracted from transgenic plants and MAC3A-HA was immunoprecipitated using anti-HA microbeads. Western blot analysis using an anti-AtCDC5 antibody revealed that AtCDC5 is present only in the eluant isolated from transgenic plants expressing MAC3A-HA and not from Col-0 control plants (Figure 6). A similar result was obtained when complementing *P35S-MAC3A-CFP* transgenic plants in the *mac3a mac3b* background were used in an independent co-IP experiment using anti-GFP microbeads (Figure S6). Thus, as in human and yeast, AtCDC5 and MAC3 associate with each other as components of the MAC in *Arabidopsis*.

## Discussion

Precursor mRNA processing is central to gene expression in eukaryotes. The three pre-mRNA processing events take place in the nucleus and include 5′ capping, 3′ polyadenylation and splicing. Splicing is orchestrated by a large ribonucleoprotein (RNP) complex called the spliceosome that produces protein-coding mRNA transcripts by splicing out introns and joining together exons. Spliceosome complexes are highly conserved and have been isolated and analyzed by proteomics approaches in budding yeast, human and *Drosophila* [22],[24],[25],[41]. Common proteins found in or associated with the spliceosome are uridine-rich snRNPs, RNA-binding proteins, RNA helicases, and serine/arginine-rich (SR) proteins. Plant spliceosome complexes have not yet been isolated, but a survey of genes in *Arabidopsis* indicates that homologs of most spliceosome proteins are conserved [34].

Several sub-complexes have been isolated along with spliceosome proteins in yeast, human, and *Drosophila*, suggesting that there are peripheral complexes that work with the core splicing machinery, perhaps to affect transcript levels and/or alternative splicing in response to environmental cues. One protein complex that associates with the spliceosome is the NTC, which was shown to act simultaneously with or just after the dissociation of the U4/U6.U5 tri-snRNP during spliceosome assembly [42]. The NTC core complex in higher eukaryotes consists of at least four proteins: SNEV/Prp19, CDC5/Cef1p, PLRG1/Prp46, and Spf27 [18], [20]–[25],[43]. It is of interest to note that *S. cerevisae* does not encode a protein with homology to MOS4/Spf27, suggesting that higher eukaryotes have evolved additional NTC proteins. Homologs of all NTC core components exist in *Arabidopsis* [34], but, like the spliceosome, this protein complex is not well understood in plants. We previously found that MOS4 (AtSpf27), AtCDC5, and PRL1 (AtPLRG1) play essential roles in plant immunity and form a nuclear protein complex called the MAC [12]. Based on what is known in other eukaryotes, we hypothesized that this protein complex corresponds to the NTC in *Arabidopsis* and likely involves more binding partners. Here we present the first effort to isolate the MAC/NTC in a plant species.

We affinity purified MOS4-HA associated proteins and isolated MAC3A and MAC3B, two *Arabidopsis* proteins with sequence homology to Prp19 (Table 1; Figure S1). This association was confirmed by co-immunoprecipitation of MAC3A-HA with AtCDC5 (Figure 6). Also found associated with MOS4-HA were AtCDC5 and PRL1, indicating that this core complex is indeed conserved in *Arabidopsis* and corroborating our previous findings [12]. A number of other predicted NTC-associated proteins were identified as well, including homologs of RBM22/Ecm2, SKIP/Prp45, Isy1, XAB2, and CRN/Clf1 (Table 1). These proteins are predicted to have roles in RNA processing in plants based on their similarity to proteins in other eukaryotes where such roles have been demonstrated [34]. However, the biological functions of these proteins have not been studied in *Arabidopsis.*

We do not yet know all the detailed protein-protein interactions within the MAC. It appears that AtCDC5 represents an interaction hub, as it physically interacts with MOS4, PRL1, and MAC3 to form the MAC core complex. We were not able to detect direct protein-protein interactions between MOS4 and MAC3A or MAC3B using either biomolecular fluorescence complementation (BiFC) or yeast-2-hybrid (data not shown). Thus, it appears that core MAC proteins form a complex through their association with AtCDC5. Tetramerization is required for Prp19 and SNEV functionality in human and yeast cells, respectively [40],[44]. This may also be the case in *Arabidopsis*, although we do not have experimental evidence for this. In yeast two-hybrid experiments, we observed neither interaction between MAC3A and MAC3B to suggest hetero-tetramerization, nor interaction between MAC3A and MAC3A or MAC3B and MAC3B to suggest homo-tetramerization (data not shown). However, these data do not rule out the possibility of MAC3 still forming homo- or hetero-tetramers. Genetic redundancy between *MAC3A* and *MAC3B* supports the idea that these proteins probably do not interact with one another to form hetero-tetramers in plants, as the loss of one or the other protein does not result in a visible phenotype. Furthermore, immunoprecipitation of MAC3A-HA followed by protein sequencing using MS did not identify MAC3B, although other core proteins such as AtCDC5 and PRL1 were identified (data not shown). We could not distinguish MAC3A from MAC3A-HA in the MS data to indicate homo-dimerization. Nonetheless, if MAC3 tetramerization is required for its function similar to Prp19 and SNEV, it is most likely that MAC3A and MAC3B form homo-tetramers in plants. Additionally, it appears that there is no preferential incorporation of MAC3A or MAC3B in the MAC, as both proteins were found to associate with MOS4-HA.

Proteins with predicted functions in splicing were also identified as MOS4-HA associated proteins, including several subunits of the U5 snRNP and one subunit of the U2 snRNP (Table 1). This is not unexpected since the NTC is known to closely associate with the spliceosome in yeast, human and *Drosophila*. The 220kD subunit of the U5 snRNP in yeast, Prp8, has recently been called “the heart of the spliceosome” [45]–[47], and forms a tri-snRNP with the ATPase Snu114p and the GTPase Brr2 (the 116kD and 200kD subunits, respectively), which are necessary for spliceosome activation. These subunits were identified as MAC components (ABNORMAL SUSPENSOR (SUS2), CLOTHO (CLO), and EMBRYO DEFECTIVE1507 (EMB1507), respectively; Table 1). Loss-of-function mutations in these genes have previously been reported to cause embryo lethality [48]–[50], agreeing with their predicted functions in splicing. However, the detailed biochemical function of these proteins has yet to be demonstrated in *Arabidopsis*.

The fact that predicted snRNP and RNA-processing proteins associate with MOS4 points to a potential role for the MAC in splicing. However, when we previously tested the fidelity of splicing machinery in *mos4-1, Atcdc5-1,* and *prl1-1* plants compared to Col-0 wild type, no difference in splicing efficiency was found for several alternatively spliced transcripts [12], suggesting that general splicing machinery is not affected by single mutations in genes encoding MAC core proteins. Also, it is unlikely that single MAC core proteins are essential for splicing because *mos4, Atcdc5, prl1,* and *mac3a mac3b* mutants are viable and have only minor morphological defects. However, when we tested genetic interactions between *mos4-1, Atcdc5-1,* and *prl1-1* by analyzing double *mos4-1 Atcdc5-1* and *mos4-1 prl1-1* mutants, we found the interactions to be synthetically lethal [12]. Synthetic lethality between MAC mutants was further confirmed when we tested the genetic interaction of *mac3a mac3b* with *mos4-1* or *prl1-1*. These triple mutants are lethal as well (data not shown). Together, these data suggest that the MAC as a whole may be required for an essential process such as spliceosome assembly, as reported for NTC components [26],[51], but that individual core MAC proteins are expendable for this process.

The yeast *prp19-1* mutant is sensitive to high temperatures and exhibits splicing defects [52]. SNEV is likewise required for spliceosome assembly in human cells [40]. Transgenic expression of full-length *SNEV* in *prp19-1* is unable to complement temperature sensitivity [40], suggesting that SNEV and Prp19 are not completely orthologous even though they share sequence homology and both exhibit E3 ligase activity [35],[36]. Similarly, we attempted to rescue *prp19-1* temperature sensitivity by expressing full-length *MAC3A* cDNA in *prp19-1*. Like SNEV, we found that expression of this *Arabidopsis* protein was unable to complement *prp19-1* in yeast (data not shown). This might indicate that yeast, human and *Arabidopsis* Prp19 may have evolved divergent biological functions, although the binding partners and enzymatic E3 ubiquitin ligase activity of these proteins seem to be conserved.

NTC proteins have been reported to be involved in many cellular processes in addition to spliceosome assembly, including DNA repair [27]–[29], cell-cycle progression [30], and protein degradation [53],[54]. We previously established a novel role for MAC proteins in plant immune signaling [12], and the data we present here for *MAC3* further highlights this. Loss-of-function *mac3a* and *mac3b* single mutant plants are not compromised in basal defense against *P.s.m.* ES4326 and *P.s.t.* DC3000, whereas *mac3a mac3b* double mutant plants exhibit enhanced susceptibility to pathogen infection, suggesting that MAC3A and MAC3B play redundant roles in plant immunity. Like *mos4-1* and *Atcdc5-1*, *mac3a mac3b* suppresses constitutive expression of *PR-1* and *PR-2*, high levels of endogenous SA, and enhanced resistance to virulent pathogens caused by *snc1*. Also, MAC3A and MAC3B seem to be required for responses mediated by specific TIR- and CC-NB-LRR R proteins. Intriguingly, MAC components appear to contribute differently to plant defense. For example, *prl1* mutants regularly sustain higher pathogen growth than *mos4, Atcdc5,* and *mac3a mac3b* mutants [12], indicating that *PRL1* may play a more prominent role in defense responses.

The *snc1* signaling network involves nucleo-cytoplasmic trafficking, RNA processing and protein modification [55]. Epistasis analysis between *snc1* and loss-of-function mutants of defense regulators defined the presence of both SA- and NPR1-independent pathways activated in *snc1* [7],[8]. Accordingly, many *MOS* genes have been shown to function in the SA-independent pathway [55]. SA accumulation following avirulent pathogen infection is unaffected in *mos4-1, Atcdc5-1,* or *prl1-1* mutants compared to Col-0 [12]. Furthermore, a double *mos4-1 npr1-1* mutant shows quantitative bacterial growth compared to *mos4-1* and *npr1-1* single mutants [12]. *MAC3A* and *MAC3B* also function in an SA-independent manner, as deduced from epistasis analysis between *eds5-3 npr1-1* and *mac3a mac3b* (data not shown). Together, these data suggest that the MAC functions independently of both SA and *NPR1*.

Plant defense responses are intimately controlled and precisely regulated in many ways including post-translational modifications resulting in protein activation, inhibition, or degradation. Targeted protein degradation in eukaryotes is mediated by a multi-protein complex called the ubiquitin proteasome [56]. This degradation is initiated by the sequential activation, conjugation, and ligation of a ubiquitin moiety to a target protein by E1, E2 and E3 enzymes, respectively [57]. One class of E3 ligase is defined by the presence of a ∼70 amino acid U-box domain [58]. U-box proteins bind both the E2 ubiquitin conjugating enzyme and the substrate to facilitate ubiquitin transfer and ligation to the target [36]. There are 64 predicted plant U-box (PUB) proteins in *Arabidopsis* and 77 in rice [59], whereas Prp19 is one of only two U-box proteins encoded in the *S. cerevisae* genome. MAC3A and MAC3B contain U-box domains (representing AtPUB59 and AtPUB60), and, like Prp19, MAC3B was recently shown to have *in vitro* E3 ubiquitin ligase activity [33], indicating that this function is conserved in plants.

The ubiquitin pathway is an integral component of *snc1* signaling. It was previously shown that the loss of the ubiquitin activating (E1) enzyme encoded by *UBA1/MOS5* (but not *UBA2,* the only other E1 enzyme in *Arabidopsis*) suppresses *snc1* autoimmune phenotypes [13]. Our finding that the loss of E3 ubiquitin ligases *MAC3A* and *MAC3B* also suppresses *snc1-*mediated responses further supports a key role for the ubiquitin pathway in *snc1* signaling. Interestingly, MAC3B appears to specifically work with two ubiquitin conjugating (E2) enzymes of the UBC4/5 class in *in vitro* ubiquitination assays [33]. This suggests that UBA1/MOS5, UBC4/5 and MAC3A/3B could potentially function together in *snc1-*mediated resistance, however this remains to be shown specifically. Although U-box proteins bind both the E2 conjugating enzyme and the substrate, we did not identify UBC4/5 (or other E2 enzymes) in the MOS4-HA pull-down. This is probably due to the transient nature of E2-E3 interactions in signal transduction pathways.

It is tempting to hypothesize that MAC3 regulates defense responses by targeting defense repressors for degradation, some of which could be identified MAC members. Several E3 ligases, including PUBs, play important roles in plant defense. The PUB E3 ligase Avr9/Cf-9 RAPIDLY ELICITED 74 (ACRE74) is required for the establishment of an HR following pathogen infection in tobacco [60], as is ACRE276 [61]. AtPUB20 and AtPUB17, the *Arabidopsis* homologs of ACRE74 and ACRE276, respectively, are up-regulated upon recognition of pathogen-derived molecules [62],[63]. Also, three redundant pathogen-induced U-box E3 ligases AtPUB22, AtPUB23 and AtPUB24 negatively regulate PAMP-triggered immunity [64]. Interestingly, the bacterial effector protein AvrPtoB mimics a eukaryotic U-box structure and has been shown to possess E3 ligase activity [65], highlighting the importance of protein degradation in plant defense signaling. Like MAC3, PRL1 is also implicated in proteasomal pathways. PRL1 inhibits the activity of AKIN10 and AKIN11, two redundant protein kinases involved in sugar signaling that interact with the α4 subunit of the 20S proteasome in *Arabidopsis* and are part of a CUL1-based E3 ubiquitin ligase complex [66]. Moreover, PRL1 was recently found to function as a substrate receptor in a CUL4-based E3 ubiquitin ligase complex [67], indicating that PRL1 plays regulatory roles in two Cullin-based E3 ligase complexes in addition to its role in the MAC.

Pathogen recognition and the activation of defense responses involves massive transcriptional reprogramming [2],[68]. The MAC core component AtCDC5 is an atypical R2R3-Myb transcription factor that binds double-stranded DNA with specificity for the element CTCAGCG [69], which is present in many *Arabidopsis* gene promoters. MOS4 is a small protein that likely serves a scaffolding function. The association between MOS4, AtCDC5, PRL1 and MAC3 suggests that both protein ubiquitination and transcriptional activation are regulatory functions employed by the MAC. As transcription is often coupled with splicing [70], it is possible that the MAC is a transcriptional modulator that functions closely with the spliceosome to regulate defense-related genes. Future studies will uncover more details about how these and other MAC proteins work together and function in plant immunity.

## Materials and Methods

### Plant growth, mutant isolation, pathology assays, and phenotypic characterization

For most experiments, plants were grown on soil in a 16h light / 8h dark regime. T-DNA mutants were obtained from the ABRC and genotyped by PCR using the insertion-flanking primers 089300-F (5′-CGGAAGTTCTTTAACTTGCGC-3′) and 089300-R (5′-GTGTTAACTGCTTCATCCGAC-3′) for *mac3a*, or 050811-F (5′-ACGGAATACTAAGCAGACCAC-3′) and 050811-R (5′-TGTTGTGCAGTGGAGTTTGATC-3′) for *mac3b*. Isolation of the *mos4-1* mutant was previously described [12]. Bacterial and oomycete infections were performed as described in [7]. Briefly, bacterial pathogens were inoculated on the abaxial leaf surfaces of four-week old plants using a needless syringe. Leaf discs (with an area of 0.38 cm^2^) were collected on the day of infection (Day 0) and three days later (Day 3) from different plants. *H.a.* isolates were spray-inoculated onto adaxial leaf surfaces of two-week old seedlings and counted using a hemocytometer seven to ten days later. RNA was extracted from 20 day old seedlings grown on Murashige and Skoog (MS) medium using the Totally RNA Kit (Ambion). Reverse transcription was performed using Superscript II reverse transcriptase (Invitrogen). The primers used to amplify *PR-1, PR-2* and *Actin 1* have been described previously [8]. *MAC3A* and *MAC3B* cDNA was amplified for expression analysis using the primers 089300RT-F (5′-CGTTGGTGACACTGATCTTG-3′), 089300RT-R (5′GCAGCAGCCGTGTAATTCAC-3′), 050811RT-F (5′-ATCTGCAGATGCGAACTCTG-3′) and 050811RT-R (5′-CCATTGCTGCAAATACTGTA-3′). SA was extracted and measured by HPLC from three-week old plants using a procedure described in [71].

### Double and triple mutant construction

To obtain the *mac3a mac3b* double mutant, homozygous *mac3a* and *mac3b* plants were crossed and the double was identified in the F_2_ using PCR-based genotyping. The *snc1 mac3a mac3b* triple mutant was obtained by crossing a homozygous *snc1* single mutant plant with a homozygous *mac3a mac3b* double mutant. *snc1* was fixed in the F_2_ first by phenotype and later confirmed by PCR using primers described previously [7]. The other two loci were confirmed by PCR to be heterozygous, and the triple mutant was identified in the F_3_. A backcross with *snc1* confirmed the genotype of the triple as all F_1_ individuals showed typical *snc1* morphology. A similar procedure was used to create the *mos4-1 mac3a mac3b* and *prl1-1 mac3a mac3b* triple mutants. All genotypes were confirmed by PCR.

### Molecular cloning

A genomic fragment spanning the full-length *MOS4* gene (*gMOS4*), without the stop codon but including a 1.5 kb sequence upstream of the translation initiation codon, was amplified from Col-0 DNA using Phusion Taq (Finnzymes) with the primers 5′-CACCACACTGCTAGAGGTCTTGG-3′, and 5′-TTGCATTTGAAGTGGCTCGAC-3′. Similarly, the open reading frames (without the stop codons) of *MAC3A* and *MAC3B* were amplified from cDNA using the primers 5′-CACCATGAATTGTGCAATTTCCGGC-3′, and 5′-TGAATCTTGTGCTGAATCTTC-3′ for *MAC3A*, and 5′-CACCATGAACTGTGCAATTTCAGGAG-3′, and 5′-TGAAATTCTCCCCCATTGCTG-3 for *MAC3B*. These Gateway-adapted PCR fragments were cloned into pENTR using the Gateway pENTR/D-Topo kit (Invitrogen). Entry vectors were confirmed by sequencing using the M13F and M13R primers. For *gMOS4,* recombination into the pGWB13 binary destination vector with a C-terminal 3xHA tag [72] was done using Gateway LR Clonase (Invitrogen). Transgenic seedlings were selected on MS plates containing 50 µg/ml Kanamycin and 50 µg/ml Hygromycin and confirmed by PCR. For *MAC3A* and *MAC3B*, recombination into destination binary vectors with a constitutive *35S* promoter for C-terminal and N-terminal fusion protein expression analysis in *Arabidopsis* and onion was done using Gateway LR Clonase (Invitrogen). Destination vectors used were either N-terminal cCFP, C-terminal cCFP, or C-terminal 3xHA-StrepII. Transgenic seedlings were selected on soil with the herbicide Basta and confirmed by PCR. Cells of transgenic seedlings grown on MS medium containing 10 mg/ml Basta were examined under the confocal microscope for CFP fluorescence as described in [10].

### Nuclear extraction and immunoprecipitation

Approximately 100 g of leaf tissue from complementing MOS4-HA in *mos4-1* was frozen in liquid nitrogen, ground into a fine powder using a pestle and mortar and homogenized in lysis buffer (20 mM Tris-HCl, pH 7.4, 25% glycerol, 20 mM KCl, 2 mM EDTA, 2.5 mM MgCl_2_, 250 mM sucrose) at 4°C. The homogenate was filtered through four layers of cheesecloth and then sequentially filtered through 100-, 70-, and 30-µm mesh nylon netting. The nuclei were pelleted by centrifugation at 1500 g for 10 minutes and washed twice with nuclei resuspension buffer (NRBT; 20 mM Tris-HCl, pH 7.4, 25% glycerol, 2.5 mM MgCl_2_, 0.5% Triton X-100). The nuclear fraction was resuspended gently in NRB and centrifuged at 100 g for 5 minutes to remove cell debris. Nuclei were pelleted by spinning the supernatant at 1500 g for 10 minutes and were resuspended in ice-cold buffer NE-2 (20 mM HEPES-KOH, pH 7.9, 2.5 mM MgCl_2_, 0.42 M NaCl, 25% glycerol, 0.2 mM EDTA, 0.5 mM DTT, with protease inhibitors). Nuclei were sonicated for 2 minutes in an ice-bath, incubated on ice for 30 minutes, and spun for 30 minutes at 25000×g at 4°C, releasing the nuclear proteins into the supernatant. The nuclear fraction was dialyzed in buffer NE-3 (20 mM HEPES-KOH, pH 7.9, 2.5 mM MgCl_2_, 100 mM KCl, 20% glycerol, 0.2 mM EDTA, 0.5 mM DTT, with protease inhibitors) for 5 hours before adding Anti-HA microbeads (Miltenyi Biotec Inc.). After gentle mixing for 30 minutes on ice, the microbeads were magnetically precipitated on columns according to the manufacturer's instructions (µMACS). The microbeads were washed eight times before elution. For identification of MOS4-HA interacting proteins, immunoprecipitated proteins were eluted and loaded onto a 12% SDS-PAGE gel and stained with a mass spectrometry-compatible silver staining kit (Invitrogen). The MOS4-HA IP lane was cut into eight pieces and digested with trypsin, and the resulting proteins were analyzed by MS (see below). To test the *in planta* interaction between MAC3A-HA and AtCDC5, a similar protein extraction procedure was employed. Approximately 15 g of leaf tissue from complementing *P35S-MAC3A-HA* in *mac3a mac3b* was frozen in liquid nitrogen and homogenized in lysis buffer as above. The homogenate was filtered through two layers of cheesecloth and sequentially filtered through 95- and 37- µm mesh nylon netting. The nuclei were pelleted as before and washed 3 times in NRBT buffer with 0.2% Triton X-100. The nuclei were then washed with NRB buffer without Triton X-100 once. Nuclei were pelleted as above and resuspended in ice-cold NE-2 buffer with the following modifications: 100 mM NaCl, 1 mM DTT, 0.2% Triton X-100. Nuclei were sonicated and pelleted as above but were not dialyzed prior to the addition of anti-HA microbeads, which were precipitated according to the manufacuter's instructions, as above. The microbeads were washed eight times prior to elution with NE-2 buffer with the following modifications: 150 mM NaCl, 1 mM DTT, 0.2% Triton X-100. The eluted fraction was loaded on a 12% SDS-PAGE gel followed by Western Blot analysis using anti-HA (Roche) or anti-AtCDC5 [12] antibodies.

### Mass spectrometry analysis

Protein bands of interest were sliced from the gel and cut into small pieces. After de-staining with ProteoSilver Distainer A and B (ProteoSilver Plus Silver Stain Kit, Sigma), the gel pieces were washed twice with ultra pure water and dehydrated in acetonitrile. The gel pieces were further dried in a CentriVap concentrator (Labconco) and then digested in-gel with 10 ng/µL sequencing grade modified trypsin (Promega) in 50 mM NH4HCO3 (pH 7.8) at 37°C overnight. The resulting tryptic peptides were extracted with 50–100 µl of 5% formic acid/50% acetonitrile and 0.1% formic acid/75% acetonitrile sequentially. The two extracts were combined and concentrated to around 10 µL in the CentriVap concentrator.

The tryptic peptides were loaded to a pre-column (75 µm×8 cm) packed with 5–15 µm spherical C18 reverse phase particles (YMC). The pre-column was connected to a home-made analytical column (50 µm×10 cm) packed with YMC 5 µm spherical C18 reverse phase particles. The tip size of the analytical column was around 2 µm and the flow rate was controlled at 20–50 nl/min. To elute peptides from the column, an Agilent 1100 series binary pumps system (Agilent Technologies) was used to generate the following HPLC gradient: 0–5% B in 5 min, 5–40% B in 25 min, 40–100% B in 10 min (A = 0.1 M acetic acid in water, B = 0.1 M acetic acid /70% acetonitrile). The eluted peptides were sprayed directly into a QSTAR XL mass spectrometer (MDS SCIEX) equipped with a nano-ESI ion source under 2.1 kV spray voltage. The mass spectra were collected in information-dependent acquisition (IDA) mode cycling with one MS scan followed by three MS/MS scans. The mass range of the MS scan was 400–2000 Da, and 100–2000 Da for MS/MS scans. For each MS scan, the top 3 most abundant precursor ions were selected for MS/MS scans using low resolution for precursor ion isolation with enhance all mode. Database searches were performed on an in-house Mascot server (version 2.1, Matrix Science Ltd.) against the NCBI non-redundant protein database.

## Supporting Information

Figure S1 Protein sequence alignment of MAC3A and MAC3B with homologs in other eukaryotes. Amino acid sequences from *Homo sapiens* (Hs; human) Prp19/SNEV (accession number NP_055317); *Mus musculus* (Mm; mouse) Prp19/SNEV (accession NP_598890); *Danio rerio* (Dr; zebra fish) Prp19 (accession number AAH45954); *Arabidopsis thaliana* (At; thale cress) MAC3A (accession number AAN13133) and MAC3B (accession number FJ820118); *Shizosaccharomyces pombe* (Sp; fission yeast) Prp19/Cwf8p (accession number CAB10135); and *Saccharomyces cerevisae* (Sc; baker’s yeast) Prp19 (accession number CAA97487), are compared. Identical amino acids are coloured black, and similar amino acids are coloured grey. Alignment was generated using ClustalW2. Boxshade version 3.21 was used to colour identical and similar amino acids. The conserved U-box and predicted nuclear localization signal (NLS) are indicated.
(0.02 MB PDF)

Figure S2 Morphology of the *mac3a mac3b* double mutant and enhanced susceptibility to *P.s.m.* ES4326. (A) Morphology of Col-0, *mac3a*, *mac3b*, and *mac3a mac3b* plants. Soil-grown plants were photographed 4 weeks after planting. Size bar represents 1 cm. (B) Growth of *P.s.m.* ES4326 at 0 and 3 days post-inoculation. Values represent an average of four replicates ± SD. This trend is apparent in several repeated experiments. These plants were grown in 16h light and low humidity, in a cooler growth chamber than is usually used for pathogen infections. An unpaired Student’s *t-*test was used to analyze the statistical significance of bacterial growth compared to Col-0. Asterisks indicate P<0.02.
(0.10 MB PDF)

Figure S3 Transgenic complementation of *mac3a mac3b* and *snc1 mac3a mac3b* by *MAC3A* and *MAC3B*. (A) Morphology of Col-0, *mac3a mac3b*, and *mac3a mac3b* plants expressing *P35S-MAC3A-CFP* or *P35S-MAC3B*. Soil-grown plants were photographed 4 weeks after planting. Size bar represents 1 cm. (B) Morphology of Col-0, *snc1*, *snc1 mac3a mac3b*, and *snc1 mac3a mac3b* plants expressing *P35S-MAC3A-CFP* or *P35S-MAC3B*. Soil-grown plants were photographed 4 weeks after planting. Bar represents 1 cm. (C) Growth of *P.s.m.* ES4326 at 0 and 3 days post-inoculation. Values represent an average of four replicates ± SD. Experiment was repeated twice with similar results. P values were calculated using an unpaired Student’s *t*-test comparing bacterial growth in *snc1 mac3a mac3b* with the transgenic lines. Asterisks indicate P<0.001.
(0.18 MB PDF)

Figure S4 Sub-cellular localization of MAC3B. Confocal microscopy was used to examine the localization of *P35S-CFP-MAC3B* in complementing transgenic *mac3a mac3b* plants. Root cells are shown. DAPI was used as a nuclear marker.
(0.01 MB PDF)

Figure S5 MAC3B cDNA sequence analysis. A comparison of *MAC3B* cDNA sequences from this study (marked with an asterisk) and from TAIR8. The error in the annotated sequence is shaded grey, as is the corrected stop codon.
(0.01 MB PDF)

Figure S6 MAC3A-CFP associates with AtCDC5 *in planta.* Total nuclear extracts were isolated from a complementing *mac3a mac3b* transgenic line expressing *P35S-MAC3A-CFP* (+) and Col-0 (-). MAC3A-CFP was immunoprecipitated using anti-GFP microbeads. MAC3A-CFP and AtCDC5 were detected in the eluted fractions by Western blot analysis using antibodies against GFP or AtCDC5. In this experiment, less input was observed in the MAC3A-CFP sample due to poor recovery of nuclei. Since less protein was present in the IP experiment, reduction in MAC3A-CFP and AtCDC5 in the flow-through is observed.
(0.05 MB PDF)

Table S1 Predicted sub-cellular localization of MAC components and details from mass spectrometry analysis. MAC proteins are organized as in Table 1, based on protein homology to NTC proteins in yeast and human. Predicted sub-cellular localization data is inferred mostly from organelle proteomics datasets, with the exception of the MAC/NTC core proteins, where localization has been shown with complementing transgenic lines expressing fluorescent protein translational fusions. Although the predicted sub-cellular localization for most MAC proteins is ambiguous, their predicted functions in spliceosome-mediated RNA processing suggest that they are nuclear. Mass spectrometry details including the number of unique peptides and sequence coverage, as well as Mascot scores, for all identified proteins, are included in the columns to the right. The 2007 version of the *Arabidopsis* genome was used for protein identification.
(0.07 MB PDF)
