# Supplementary material for: Two Prp19-Like U-Box Proteins in the MOS4-Associated Complex Play Redundant Roles in Plant Innate Immunity
Source: PLoS Pathog. 2009 Jul 24;5(7):e1000526. doi: 10.1371/journal.ppat.1000526 (PMC2709443; doi:10.1371/journal.ppat.1000526)
Supplement: Figure S1 — Protein sequence alignment of MAC3A and MAC3B with homologs in other eukaryotes. Amino acid sequences from Homo sapiens (Hs; human) Prp19/SNEV (accession number NP_055317); Mus musculus (Mm; mouse) Prp19/SNEV (accession NP_598890); Danio rerio (Dr; zebra fish) Prp19 (accession number AAH45954); Arabidopsis thaliana (At; thale cress) MAC3A (accession number AAN13133) and MAC3B (accession number FJ820118); Shizosaccharomyces pombe (Sp; fission yeast) Prp19/Cwf8p (accession number CAB10135); and Saccharomyces cerevisae (Sc; baker’s yeast) Prp19 (accession number CAA97487), are compared. Identical amino acids are coloured black, and similar amino acids are coloured grey. Alignment was generated using ClustalW2. Boxshade version 3.21 was used to colour identical and similar amino acids. The conserved U-box and predicted nuclear localization signal (NLS) are indicated. (0.02 MB PDF) [file ppat.1000526.s001.pdf]

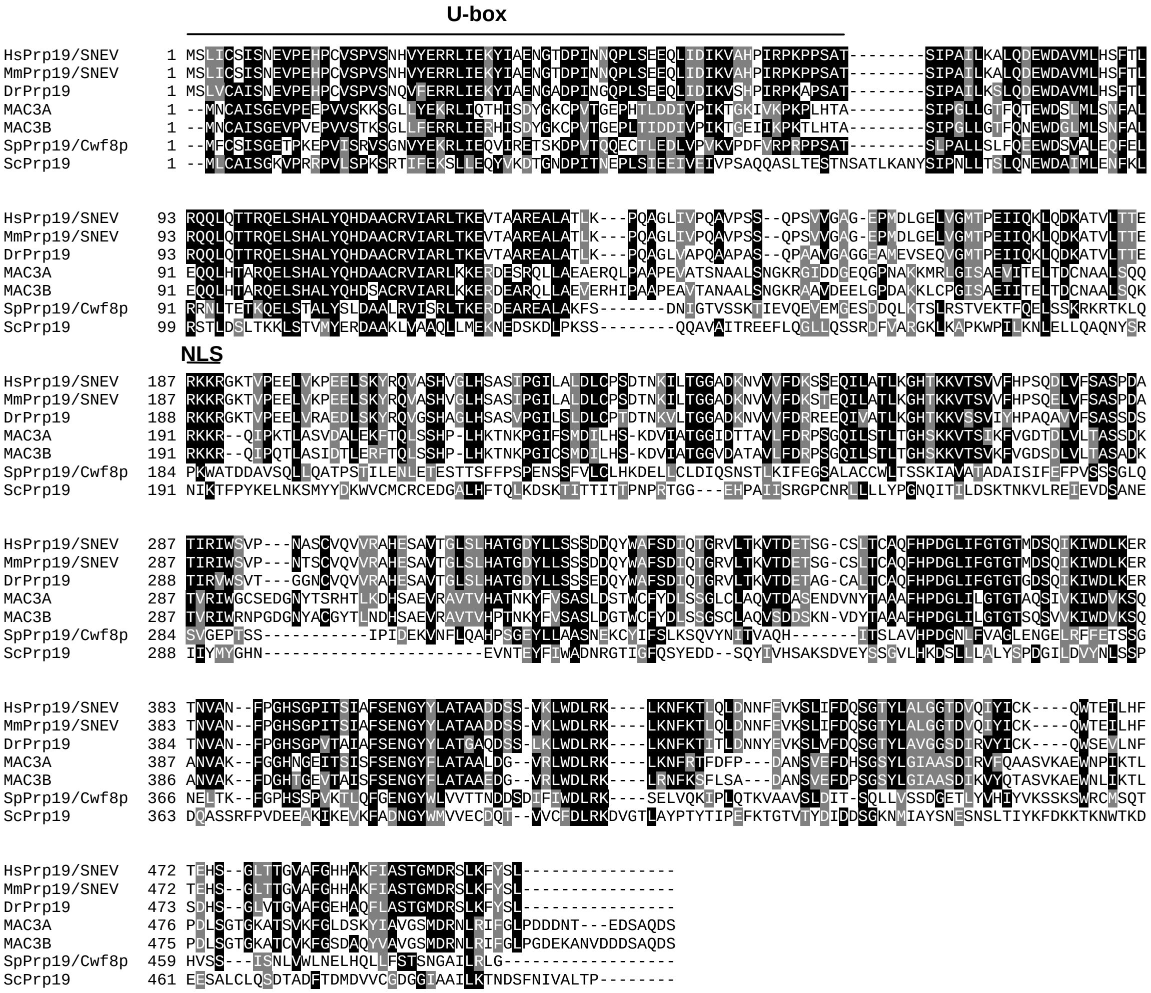

Figure S1. Protein sequence alignment of MAC3A and MAC3B with homologs in other eukaryotes.

Amino acid sequences from *Homo sapiens* (Hs; human) Prp19/SNEV (accession number NP_055317); *Mus musculus* (Mm; mouse) Prp19/SNEV (accession NP_598890); *Danio rerio* (Dr; zebra fish) Prp19 (accession number AAH45954); *Arabidopsis thaliana* (At; thale cress) MAC3A (accession number AAN13133) and MAC3B (accession number FJ820118); *Shizosaccharomyces pombe* (Sp; fission yeast) Prp19/Cwf8p (accession number CAB10135); and *Saccharomyces cerevisae* (Sc; baker's yeast) Prp19 (accession number CAA97487), are compared. Identical amino acids are coloured black, and similar amino acids are coloured grey. Alignment was generated using ClustalW2. Boxshade version 3.21 was used to colour identical and similar amino acids. The conserved U-box and predicted nuclear localization signal (NLS) are indicated.
