# Supplementary material for: Two Prp19-Like U-Box Proteins in the MOS4-Associated Complex Play Redundant Roles in Plant Innate Immunity
Source: PLoS Pathog. 2009 Jul 24;5(7):e1000526. doi: 10.1371/journal.ppat.1000526 (PMC2709443; doi:10.1371/journal.ppat.1000526)
Supplement: Figure S2 — Morphology of the mac3a mac3b double mutant and enhanced susceptibility to P.s.m. ES4326. (A) Morphology of Col-0, mac3a, mac3b, and mac3a mac3b plants. Soil-grown plants were photographed 4 weeks after planting. Size bar represents 1 cm. (B) Growth of P.s.m. ES4326 at 0 and 3 days post-inoculation. Values represent an average of four replicates ± SD. This trend is apparent in several repeated experiments. These plants were grown in 16h light and low humidity, in a cooler growth chamber than is usually used for pathogen infections. An unpaired Student’s t-test was used to analyze the statistical significance of bacterial growth compared to Col-0. Asterisks indicate P<0.02. (0.10 MB PDF) [file ppat.1000526.s002.pdf]

A

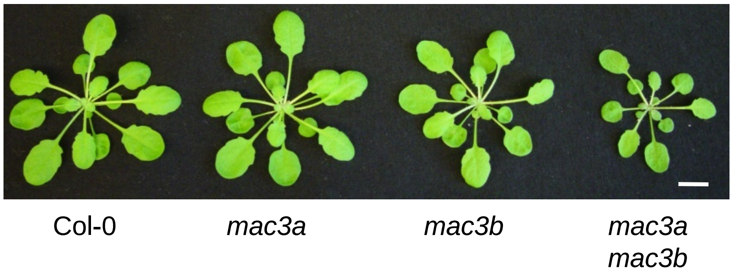

B

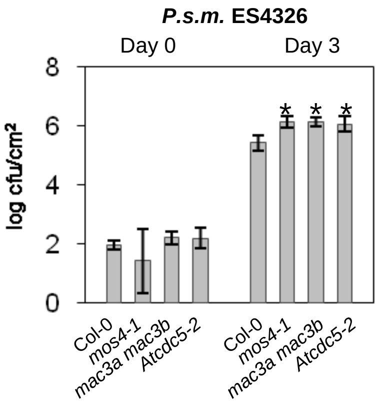

Figure S2. Morphology of the *mac3a mac3b* double mutant and enhanced susceptibility to *P.s.m.* ES4326.

(A) Morphology of Col-0, *mac3a*, *mac3b*, and *mac3a mac3b* plants. Soil-grown plants were photographed 4 weeks after planting. Size bar represents 1 cm. (B) Growth of *P.s.m.* ES4326 at 0 and 3 days post-inoculation. Values represent an average of four replicates ± SD. This trend is apparent in several repeated experiments. These plants were grown in 16h light and low humidity, in a cooler growth chamber than is usually used for pathogen infections. An unpaired Student's *t*-test was used to analyze the statistical significance of bacterial growth compared to Col-0. Asterisks indicate P<0.02.
