# Supplementary material for: Two Prp19-Like U-Box Proteins in the MOS4-Associated Complex Play Redundant Roles in Plant Innate Immunity
Source: PLoS Pathog. 2009 Jul 24;5(7):e1000526. doi: 10.1371/journal.ppat.1000526 (PMC2709443; doi:10.1371/journal.ppat.1000526)
Supplement: Figure S3 — Transgenic complementation of mac3a mac3b and snc1 mac3a mac3b by MAC3A and MAC3B. (A) Morphology of Col-0, mac3a mac3b, and mac3a mac3b plants expressing P35S-MAC3A-CFP or P35S-MAC3B. Soil-grown plants were photographed 4 weeks after planting. Size bar represents 1 cm. (B) Morphology of Col-0, snc1, snc1 mac3a mac3b, and snc1 mac3a mac3b plants expressing P35S-MAC3A-CFP or P35S-MAC3B. Soil-grown plants were photographed 4 weeks after planting. Bar represents 1 cm. (C) Growth of P.s.m. ES4326 at 0 and 3 days post-inoculation. Values represent an average of four replicates ± SD. Experiment was repeated twice with similar results. P values were calculated using an unpaired Student’s t-test comparing bacterial growth in snc1 mac3a mac3b with the transgenic lines. Asterisks indicate P<0.001. (0.18 MB PDF) [file ppat.1000526.s003.pdf]

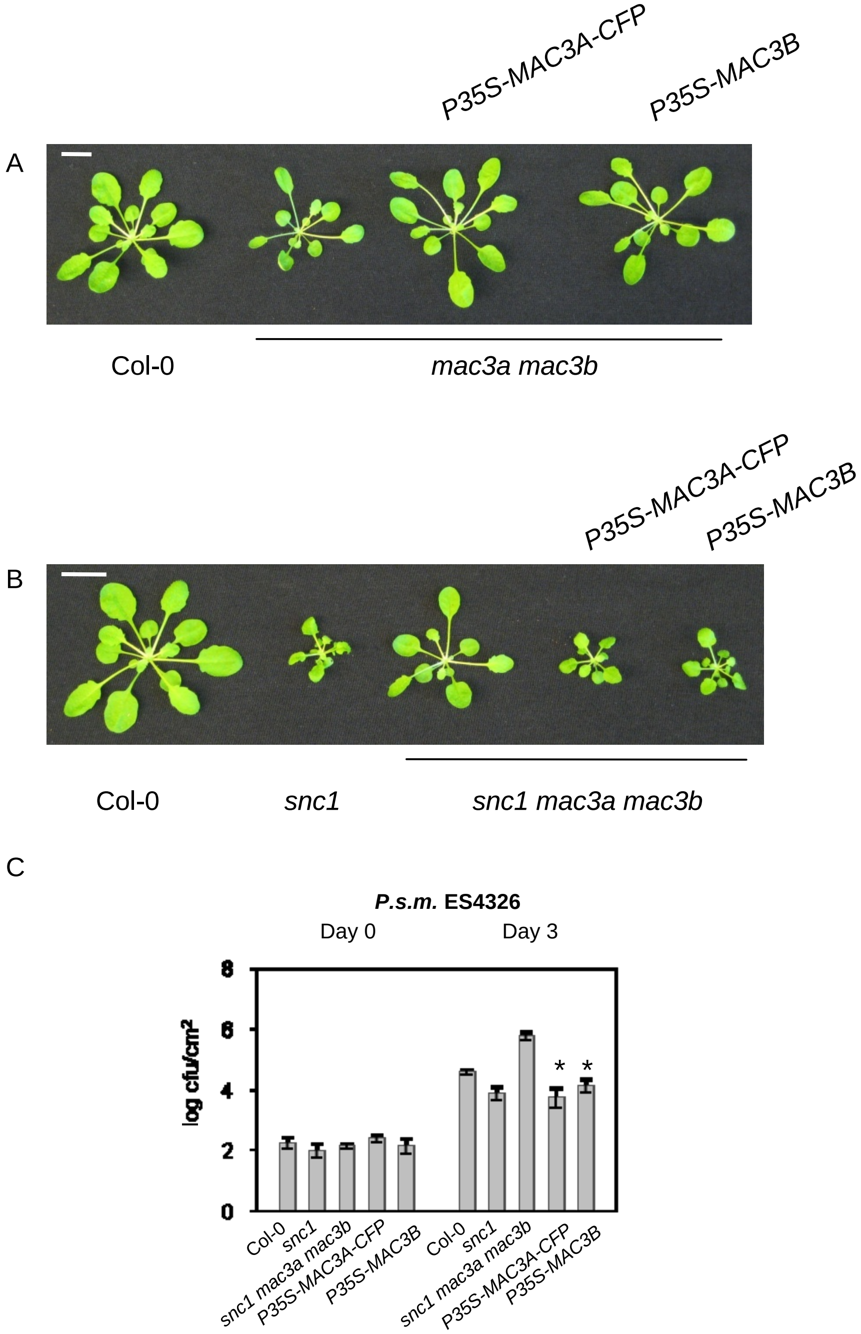

Figure S3. Transgenic complementation of *mac3a mac3b* and *snc1 mac3a mac3b* by *MAC3A* and *MAC3B*.

(A) Morphology of Col-0, *mac3a mac3b*, and *mac3a mac3b* plants expressing *P35S-MAC3A-CFP* or *P35S-MAC3B*. Soil-grown plants were photographed 4 weeks after planting. Size bar represents 1 cm. (B) Morphology of Col-0, *snc1*, *snc1 mac3a mac3b*, and *snc1 mac3a mac3b* plants expressing *P35S-MAC3A-CFP* or *P35S-MAC3B*. Soil-grown plants were photographed 4 weeks after planting. Bar represents 1 cm. (C) Growth of *P.s.m.* ES4326 at 0 and 3 days post-inoculation. Values represent an average of four replicates ± SD. Experiment was repeated twice with similar results. P values were calculated using an unpaired Student's *t*-test comparing bacterial growth in *snc1 mac3a mac3b* with the transgenic lines. Asterisks indicate $P<0.001$.
