# Supplementary material for: Two Prp19-Like U-Box Proteins in the MOS4-Associated Complex Play Redundant Roles in Plant Innate Immunity
Source: PLoS Pathog. 2009 Jul 24;5(7):e1000526. doi: 10.1371/journal.ppat.1000526 (PMC2709443; doi:10.1371/journal.ppat.1000526)
Supplement: Figure S4 — Sub-cellular localization of MAC3B. Confocal microscopy was used to examine the localization of P35S-CFP-MAC3B in complementing transgenic mac3a mac3b plants. Root cells are shown. DAPI was used as a nuclear marker. (0.01 MB PDF) [file ppat.1000526.s004.pdf]

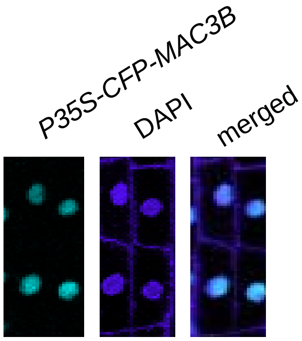

Figure S4. Sub-cellular localization of MAC3B.

Confocal microscopy was used to examine the localization of *P35S-CFP-MAC3B* in complementing transgenic *mac3a mac3b* plants. Root cells are shown. DAPI was used as a nuclear marker.
