# Supplementary material for: Two Prp19-Like U-Box Proteins in the MOS4-Associated Complex Play Redundant Roles in Plant Innate Immunity
Source: PLoS Pathog. 2009 Jul 24;5(7):e1000526. doi: 10.1371/journal.ppat.1000526 (PMC2709443; doi:10.1371/journal.ppat.1000526)
Supplement: Figure S5 — MAC3B cDNA sequence analysis. A comparison of MAC3B cDNA sequences from this study (marked with an asterisk) and from TAIR8. The error in the annotated sequence is shaded grey, as is the corrected stop codon. (0.01 MB PDF) [file ppat.1000526.s005.pdf]

```
                                                                                 Stop
               D  R  N  L  R  I  F  G  L  P  G  D  E  K  A  N  V  D  D  D  S  A  Q  D  S  -     525
MAC3B*        GACCGTAACCTACGGATATTTGGTCTTCCTGGTGATGAAAAAGCCAACGTCGATGATGACTCTGCGCAAGACTCGTGA-
TAIR          GACCGTAAC-TACGGATATTTGGTCTTCCTGGTGATGAAAAAGCCAACGTCGATGATGACTCTGCGCAAGACTCGTGAA
               D  R  N   Y  G  Y  L  V  F  L  V  M  K  K  P  T  S  M  M  T  L  R  K  T  R  E    526

               -  -  -  -  -  -  -  -  -  -  -  -  -  -  -  -  -  -  -  -  -  -  -  -  -  -  -
MAC3B*        ---------------------------------------------------------------------------------
TAIR          CAATCCGTAAAAAACTCAGACAGAACATTCCAAATGTCCATCTCCAGTGTTATCTCGCTCAAATTTCTCAAGTTCTGTTGT
               Q  S  V  K  N  S  D  R  T  F  Q  M  S  I  S  S  V  I  S  L  K  F  L  K  F  C  C  553

               -  -  -  -  -  -  -  -  -  -  -  -  -
MAC3B*        ---------------------------------------
TAIR          ATCTTACAGTATTTGCAGCAATGGGGGAGAATTTCATAG
               I  L  Q  Y  L  Q  Q  W  G  R  I  S  -  565
```

Figure S5. MAC3B cDNA sequence analysis.

A comparison of *MAC3B* cDNA sequences from this study (marked with an asterisk) and from TAIR8. The error in the annotated sequence is shaded grey, as is the corrected stop codon.
