# Supplementary material for: Two Prp19-Like U-Box Proteins in the MOS4-Associated Complex Play Redundant Roles in Plant Innate Immunity
Source: PLoS Pathog. 2009 Jul 24;5(7):e1000526. doi: 10.1371/journal.ppat.1000526 (PMC2709443; doi:10.1371/journal.ppat.1000526)
Supplement: Figure S6 — MAC3A-CFP associates with AtCDC5 in planta. Total nuclear extracts were isolated from a complementing mac3a mac3b transgenic line expressing P35S-MAC3A-CFP (+) and Col-0 (-). MAC3A-CFP was immunoprecipitated using anti-GFP microbeads. MAC3A-CFP and AtCDC5 were detected in the eluted fractions by Western blot analysis using antibodies against GFP or AtCDC5. In this experiment, less input was observed in the MAC3A-CFP sample due to poor recovery of nuclei. Since less protein was present in the IP experiment, reduction in MAC3A-CFP and AtCDC5 in the flow-through is observed. (0.05 MB PDF) [file ppat.1000526.s006.pdf]

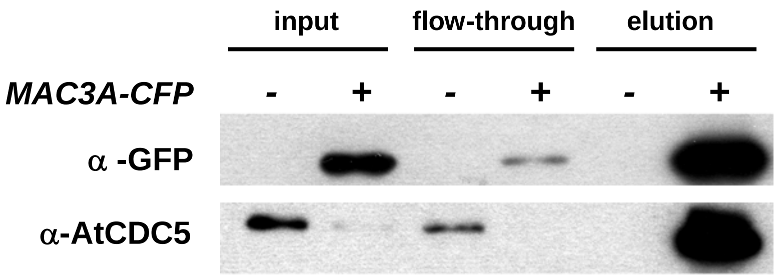

Figure S6. MAC3A-CFP associates with AtCDC5 *in planta.*

Total nuclear extracts were isolated from a complementing *mac3a mac3b* transgenic line expressing *P35S-MAC3A-CFP* (+) and Col-0 (-). MAC3A-CFP was immunoprecipitated using anti-GFP microbeads. MAC3A-CFP and AtCDC5 were detected in the eluted fractions by Western blot analysis using antibodies against GFP or AtCDC5. In this experiment, less input was observed in the MAC3A-CFP sample due to poor recovery of nuclei. Since less protein was present in the IP experiment, reduction in MAC3A-CFP and AtCDC5 in the flow-through is observed.
