# Supplementary material for: Two Prp19-Like U-Box Proteins in the MOS4-Associated Complex Play Redundant Roles in Plant Innate Immunity
Source: PLoS Pathog. 2009 Jul 24;5(7):e1000526. doi: 10.1371/journal.ppat.1000526 (PMC2709443; doi:10.1371/journal.ppat.1000526)
Supplement: Table S1 — Predicted sub-cellular localization of MAC components and details from mass spectrometry analysis. MAC proteins are organized as in Table 1, based on protein homology to NTC proteins in yeast and human. Predicted sub-cellular localization data is inferred mostly from organelle proteomics datasets, with the exception of the MAC/NTC core proteins, where localization has been shown with complementing transgenic lines expressing fluorescent protein translational fusions. Although the predicted sub-cellular localization for most MAC proteins is ambiguous, their predicted functions in spliceosome-mediated RNA processing suggest that they are nuclear. Mass spectrometry details including the number of unique peptides and sequence coverage, as well as Mascot scores, for all identified proteins, are included in the columns to the right. The 2007 version of the Arabidopsis genome was used for protein identification. (0.07 MB PDF) [file ppat.1000526.s007.pdf]

Table S1. Predicted sub-cellular localization of MAC components and details from mass spectrometry analysis.

		Predicted subcellular localization (TAIR version 8)			Mass Spectrometry Details			
At number	Protein description	Localization	Experimental evidence	Reference (below)	GI	Mascot score**	Number of unique peptide matches (score > 20)	Sequence Coverage (%)
NTC Core Proteins*								
AT1G09770	AtCDC5/MAC1; R2-R3 Myb transcription factor	Nucleus	GFP-fusion, stably transformed	1	1747310	1381	25	35
AT1G04510	MAC3A; PUB; WD-40 repeat family	Nucleus	CFP-fusion, stably transformed	this work	18390401	816	12	29
AT2G33340	MAC3B; PUB; WD-40 repeat family	Nucleus	CFP-fusion, stably transformed	this work	2459417	991	18	37
AT3G18165	MOS4; protein-protein interactions	Nucleus	GFP-fusion, stably transformed	2	9294072	324	7	20
AT4G15900	PRL1/MAC2; WD-40 repeat family	Nucleus	GFP-fusion, stably transformed	2	15234752	719	12	32
Other NTC Associated Proteins*								
AT1G07360	zinc finger (CCCH-type) family protein; RNA binding	Unknown	N/A	N/A	15450878	314	5	11
AT1G77180	chromatin protein family	Nucleolus, nucleus	organelle proteome datasets	3,4	18411393	937	20	34
AT2G38770	EMB2765; helicase	Chloroplast, plasma membrane	organelle proteome datasets	5,6	3785995	1650	33	26
AT3G18790	similar to two coiled coil domains of eukaryotic ori	Chloroplast	organelle proteome datasets	5	15230193	289	7	28
AT5G28740	transcription-coupled DNA repair-related; RNA processing	Unknown	N/A	N/A	15241911	973	23	25
AT5G41770	cell cycle control crooked neck protein-like; RNA processing	Unknown	N/A	N/A	25083215	1428	31	40
Predicted splicing-related proteins*								
AT1G06220	CLO/GFA1/MEE5; translation elongation; nucleic acid binding	Cell wall, plasma membrane	organelle proteome datasets	7,8	15221423	2389	43	51
AT1G10580	WD-40 repeat family; nucleotide binding	Unknown	N/A	N/A	5091552	1067	21	33
AT1G15200	protein-protein interaction regulator family	Unknown	N/A	N/A	18394209	648	15	34
AT1G20960	EMB1507; DEAD-box helicase	membrane, nucleolus	organelle proteome datasets	4,9	15218086	4805	94	49
AT1G32490	EMB2733/ESP3; DEAD-box like helicase	endomembrane	TargetP1.1	N/A	8920625	384	11	13
AT1G80070	SUS2/EMB177; embryogenesis	chloroplast, membrane, nucleus	organelle proteome, Interpro	5,8	5902365	4973	105	48
AT2G43770	WD-40 repeat family; nucleotide binding	Unknown	N/A	N/A	15224356	630	10	29
AT5G64270	putative splicing factor similar to RCN1; PP2A regulator	chloroplast	organelle proteome datasets	5	15237657	440	10	7
Unrelated proteins*								
AT3G15730	PLDα1; hormone response	chloroplast, membrane	organelle proteome datasets	5,8,10	15232671	1318	26	42
AT3G20820	LRR family; defense response	cell wall, membrane, chloroplast, apoplast	organelle proteome datasets	5,6,11,12	15232373	736	11	37
AT3G60190	ADL1E/DRP1E/EDR3; GTPase; defense response	plasma membrane, vacuole	organelle proteome datasets	6,13,14	18411520	609	13	26
AT4G19410	putative pectin acetylesterase; carboxylesterase activity	cell wall, membrane	organelle proteome datasets	7,8,14	18415308	315	6	16
AT5G42080	ADL1A/DRP1A/RSW9; GTPase	plasma membrane, vacuole	organelle proteome datasets	6,13,14	30693985	1290	27	41

* As organized in Wang BB, Brendel V (2004) The ASRG database: identification and survey of Arabidopsis thaliana genes involved in pre-mRNA splicing. Genome Biol 5: R102.

** Mascot calcuates protein score as $-10*LOG_{10}(P)$, where P is the absolute probability that the match is a random event.

MAC proteins are organized as in Table 1, based on protein homology to NTC proteins in yeast and human. Predicted sub-cellular localization data is inferred mostly from organelle proteomics datasets, with the exception of the MAC/NTC core proteins, where localization has been shown with complementing transgenic lines expressing fluorescent protein translational fusions. Although the predicted sub-cellular localization for most MAC proteins is ambiguous, their predicted functions in spliceosome-mediated RNA processing suggest that they are nuclear. Mass spectrometry details including the number of unique peptides and sequence coverage, as well as Mascot scores, for all identified proteins, are included in the columns to the right. The 2007 version of the *Arabidopsis* genome was used for protein identification.

References

1 Lin Z, Yin K, Wang X, Liu M, Chen Z, et al. (2007) Virus induced gene silencing of AtCDC5 results in accelerated cell death in Arabidopsis leaves. Plant Physiol Biochem 45: 87-94.
2 Palma K, Zhao Q, Cheng YT, Bi D, Monaghan J, et al. (2007) Regulation of plant innate immunity by three proteins in a complex conserved across the plant and animal kingdoms. Genes Dev 21: 1484-1493.
3 Pendle AF, Clark GP, Boon R, Lewandowska D, Lam Y, et al. (2004) Proteomic Analysis of the Arabidopsis Nucleolus Suggests Novel Nucleolar Functions. Mol Biol Cell 16: 260-269.
4 Bae MS, Cho EJ, Choi EY, Park OK. (2003) Analysis of the Arabidopsis nuclear proteome and its response to cold stress. Plant J 36: 652-63.
5 Kleffmann T, Russenberger D, von Zychlinski A, Christopher W, Sjolander K, et al. (2004) The Arabidopsis thaliana chloroplast proteome reveals pathway abundance and novel protein functions. Curr Biol 14: 354-62.
6 Benschop JJ, Mohammed S, O'Flaherty M, Heck AJR, Slijper M, et al. (2007) Quantitative phospho-proteomics of early elicitor signalling in Arabidopsis. Mol Cell Proteomics6:1198-1214
7 Bayer EM, Bottrill AR, Walshaw J, Vigouroux M, Naldrett M, et al. (2005) Arabidopsis cell wall proteome defined using multidimensional protein identification technology. Proteomics 6: 301-11.
8 Mitra S, Gantt J, Ruby J, Clouse S, Goshe M. (2007) Membrane Proteomic Analysis of Arabidopsis thaliana Using Alternative Solubilization Techniques. J Proteome Res 6: 1933-1950.
9 Zybailov B, Rutschow H, Friso G, Rudella A, Emanuelsson O, et al. (2008) Sorting signals, N-terminal modifications and abundance of the chloroplast proteome. PLoS ONE 3: e1994.
10 Boudart G, Jamet E, Rossignol M, Lafitte C, Borderies G, et al. (2005) Cell wall proteins in apoplastic fluids of Arabidopsis thaliana rosettes: identification by mass spectrometry and bioinformatics. Proteomics 5: 212-221.
11 Bindschedler LV, Palmblad M, Cramer R. (2008) Hydroponic isotope labelling of entire plants (HILEP) for quantitative plant proteomics; an oxidative stress case study. Phytochemistry 69: 1962-72.
12 Carter C, Pan S, Zouhar J, Avila EL, Girke T, et al. (2004) The vegetative vacuole proteome of Arabidopsis thaliana reveals predicted and unexpected proteins. Plant Cell 16: 3285-3303.
13 Jaquinod M, Villiers F, Kieffer-Jaquinod S, Hugouvieux V, Bruley C, et al. (2006) A proteomic dissection of Arabidopsis thaliana vacuoles isolated from cell culture. Mol Cell Proteomics 6: 394-412.
14 Borderies G, Jamet E, Lafitte C, Rossignol M, Jauneau A, et al. (2003) Proteomics of loosely bound cell wall proteins of Arabidopsis thaliana cell suspension cultures: a critical analysis. Electrophoresis 24: 3421-32.
